# Prevalence and antibiotic susceptibility patterns of enteric bacterial pathogens in human and non-human sources in an urban informal settlement in Cape Town, South Africa

**DOI:** 10.1186/s12866-019-1620-6

**Published:** 2019-11-06

**Authors:** John Bosco Kalule, Anthony M. Smith, Mjikisile Vulindhlu, Nomsa P. Tau, Mark P. Nicol, Karen H. Keddy, Lourens Robberts

**Affiliations:** 1 Division of Medical Microbiology, Department of Pathology, Faculty of Health Sciences, University of Cape Town and National Health Laboratory Services, Cape Town, South Africa; 2 Centre for Enteric Diseases, National Institute for Communicable Diseases, Johannesburg, South Africa; 3 Faculty of Health Sciences, University of the Witwatersrand, Johannesburg, South Africa; 4 Cape Town Water and Sanitation Department, City of Cape Town, South Africa

**Keywords:** Informal settlement, Foodborne pathogens, Antibiograms, Diarrhoea

## Abstract

**Background:**

In light of rampant childhood diarrhoea, this study investigated bacterial pathogens from human and non-human sources in an urban informal settlement.

Meat from informal abattoirs (*n* = 85), river water (*n* = 64), and diarrheic stool (*n* = 66) were collected between September 2015 and May 2016. A duplex real-time PCR, gel-based PCR, and CHROMagar™STEC were used to screen Tryptic Soy Broth (TSB) for diarrheic *E. coli*. Standard methods were used to screen for other selected food and waterborne bacterial pathogens.

**Results:**

Pathogens isolated from stool, meat, and surface water included *Salmonella enterica* (6, 5, 0%), *Plesiomonas shigelloides* (9, 0, 17%), *Aeromonas sobria* (3, 3, 0%), *Campylobacter jejuni* (5, 5, 0%), *Shigella flexneri* (17, 5, 0%), *Vibrio vulnificus* (0, 0, 9%), and diarrheic *E. coli* (21, 3, 7%) respectively. All the isolates were resistant to trimethoprim–sulphamethoxazole.

**Conclusions:**

There was a high burden of drug resistant diarrheal pathogens in the stool, surface water and meat from informal slaughter. Integrated control measures are needed to ensure food safety and to prevent the spread of drug resistant pathogens in similar settings.

## Background

Diarrheal disease is a major cause of morbidity in the Western Cape province and the city of Cape Town in South Africa [1]. Infant mortality from diarrhea in South Africa is characterized by a seasonal unimodal peak from March – June each year [2]. In the Western Cape and Cape Town, the peak is experienced around March [3]. A recent South African Demographic and Health Survey (2016) showed that 10% of children under five years had experienced diarrhea in the preceding two weeks; 63% of these sought medical treatment [4]. Diarrhea is the second leading cause of under-five mortality in South Africa (16%), trailing behind HIV/AIDS (20.1%) [5]. In the Western Cape Province, diarrhea is the 3rd leading cause of under-five mortality (11%); almost half (42.9%) of child diarrheal deaths in the Cape Town metro sub-district occur at home [3]. Among those that seek primary healthcare, some are locally managed, while others are referred to tertiary care; a main contributing factor for diarrheal death among those referred to tertiary care in South Africa is a failure to correctly assess the severity of the child’s condition at the primary care level [6]. Of those hospitalized, the under 5 case fatality rate attributable to diarrhea is estimated at 7.3% [6].

Public primary health care practice in South Africa relies on the syndromic management of diarrheal disease [7]. The South African Standard Treatment Guidelines (STGs) for acute diarrhoea in children recommend rehydration and a single dose of intramuscular ceftriaxone at 80 mg/kg for infants (< 4 weeks old), the malnourished, and where danger signs exist. Explicitly, for dysentery, the STGs indicate treatment with oral ciprofloxacin (15 mg/kg 12 hourly for three days) followed by treatment for amoebic dysentery if symptoms do not improve after three days [8].

Supranational surveillance projects such as the Global Enteric Multicenter Study (GEMS) [9] have provided relevant information on the common causes of paediatric infectious diarrhea in sub-Saharan Africa but demonstrated that public health interventions should be based on locally generated data [10]. A better understanding of the local environmental factors that favour transmission of enteric pathogens is particularly crucial for vulnerable communities such as those living in poverty in low-cost housing settlements [11]. Given the unhygienic conditions at local abattoirs, improper disposal of abattoir and domestic waste, and the reports of childhood diarrhea, we set out to identify the enteric bacterial pathogens present in an urban informal settlement in Cape Town. We therefore screened children that presented with diarrhea at the local Community Health Center, water from the local canal, and meat from the local informal food trade.

## Methods

### Setting

**Fig. 1:**
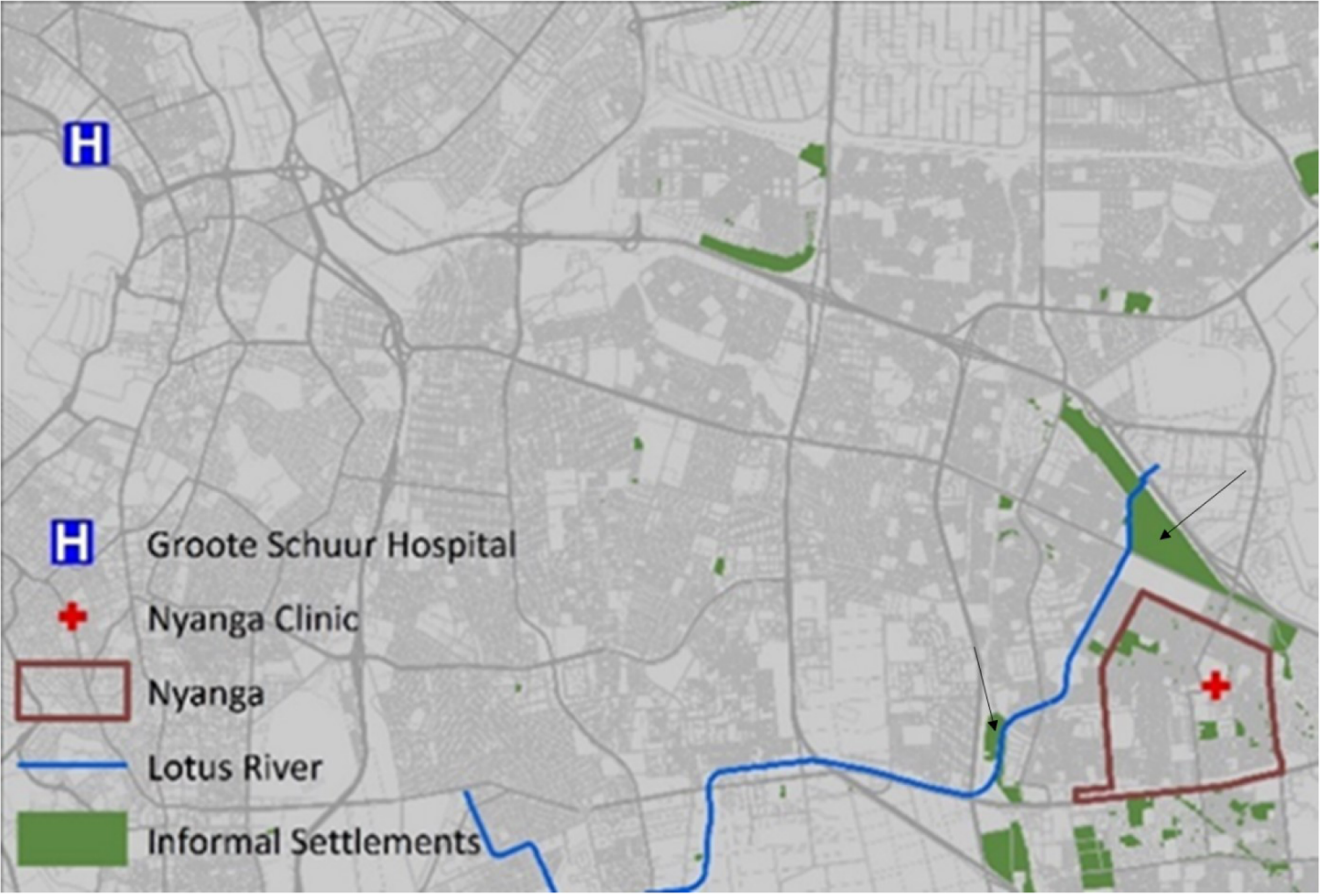
Showing the location of the informal settlement, the Nyanga clinic, and the Lotus River. The black arrows indicate the areas where the meat samples were collected. This image was created using Arc GIS version 10.5

The City of Cape Town is temperate with moderately wet winters and dry, warm summers. The Nyanga urban informal settlement surveyed is comprised of ~ 16,000 dwellings with ~ 58,000 residents. The population density is estimated at 19,000/km^2^. Eighty-one percent of households have access to communal flush toilets connected to a sewer system, while 11% rely on bucket toilets; and 3% report no access to toilet facilities. Only 53.5% of households have piped water inside their dwellings [12]. The Nyanga settlement is located in a seasonal wetland that has been canalized subsequent to urbanization resulting in a canalized river flowing through this densely populated community (Fig. 1). Informal abattoirs are an important component of the micro-economy and include the street-side slaughter of livestock and the selling of meat and meat products.

### Patient selection and specimen sampling

The caregivers of children (under 12 years of age) with diarrhoea, attending the local Community Health Center (CDC) on Monday, Wednesday, and Friday of every week between October 2015 and April 2016, were approached at random for enrolment after informed consent was obtained. Clinical and epidemiological data was collected from consenting caregivers. Freshly passed stool was collected using a screw-capped stool collection container (Sarstedt, Nümbrecht, Germany) from the diaper (or a triple tissue layer). Meat samples (raw and roasted chicken, beef, and pork) from all road-side stalls in the community were collected randomly in sterile stomacher bags (Tekmar, Cincinnati, OH) on the first two Fridays of every month using the guidelines for integrated surveillance of foodborne antimicrobial resistance [13]. Surface water samples were collected from four points (LR13, LR14, LR15, and LR16) once every two weeks for eight months along the canalized river using the depth-integrated grab sampling method at a depth of 30 cm below the surface towards the center of the stream [14]. Samples were transported to the laboratory within 12 h of the collection in a 4 °C temperature – monitored box.

This non-interventional survey study was approved by the Human Subjects Research Ethics Committee of the University of Cape Town (HREC 2015/140) and the City of Cape Town Health Department, Klipfontein Health sub-district (6547–10,516).

Informed consent: “Informed consent was obtained from all individual participants included in this study.

### Sample processing

A pea-sized amount of stool, 25 g of meat tissue (pummeled using a sterile mortar and pestle), and the filtrate (on a 0.45 μm membrane filter) of 100 ml of surface water were inoculated in 90 ml, 225 ml, and 90 ml of TSB (Oxoid, Basingstoke, UK) respectively and incubated at 35 °C overnight as previously described [15, 16]. From overnight enrichments, a loopful was streaked on CHROMagar™STEC (CHROMagar Microbiology, Paris, France) for STEC, Xylose Lysine Deoxycholate (XLD, Greenpoint Media Laboratory, NHLS, Albertynshof) for *Shigella* and *Salmonella,* and Cefsulodin -Irgasan-Novobiocin (CIN, Greenpoint Media Laboratory, NHLS, Albertynshof) for *Aeromonas* and *Yersinia* with overnight incubation at 35 °C. For *Campylobacter,* samples (1 ml of surface water, 1 ml of tissue rinsate in TSB, or a loopful of stool sample) were directly streaked onto Charcoal Cefoperazone Deoxycholate modified Agar (CCDA) and incubated under microaerophilic conditions for 72 h at 35 °C. To culture *Vibrio*, 10 ml of water or 1-10 ml of stool was inoculated in 90 ml of Alkaline Peptone Water and incubated at 42 °C for eight hours. Subsequently, a loopful of the surface pellicle was sub-cultured onto a Thiosulfate – Citrate – Bile salts – Sucrose (TCBS) agar and incubated overnight at 35 °C. For P. *shigelloides*, a loopful of TSB was used to inoculate MacConkey agar with crystal violet (McA) and Cefsulodin – Irgasan - Novobiocin (CIN) agar and incubated at 35 °C for 24 h. Non- lactose fermenting colonies on McA and opaque colonies with a pink center on CIN, were then tested for oxidase activity before confirmatory identification. A maximum of five mauve colonies from each CHROMagar™STEC plate were sub-cultured separately on McA agar with crystal violet and sorbitol MacConkey agar plates. Suspect colonies from the different selective media were picked for confirmatory identification and characterisation. All isolates were identified, and their minimum inhibitory concentration (MIC) values determined using VITEK 2 (bioMérieux, USA). *E. coli* isolates from CHROMagar™STEC were tested for the presence of *stx* using real-time PCR on a LightCycler®480 II Instrument (Roche Life Sciences, Industriestrasse, Switzerland) using LightCycler® 480 Probes Master mastermix and primers as previously described. (Table 1) [18].

**Table 1:** Primers and probes for the duplex real-time PCR assay

Primer/probe	5́ Dye	Sequence	3́ Dye	Reference
*stx*_*1*_a-primer		CAAGAGCGATGTTACGGT		[18]
*stx*_*1*_b-primer		AATTCTTCCTACACGAACAGA		[17]
*stx*_*1*_f-probe		CTGGGGAAGGTTGAGTAGCG	Fluorescein	[17]
*stx*_*1*_r-probe	CALFluor 610	CCTGCCTGACTATCATGGACA	3′ phosphor	[17]
*stx*_*2*_a-primer		GGGACCACATCGGTGT		[17]
*stx*_*2*_b-primer		CGGGCACTGATATATGTGTAA		[17]
*stx*_*2*_f-probe		CTGTGGATATACGAGGGCTTGATGTC	Fluorescein	[17]
stx_2_r-probe	CAL Fluor 610	ATCAGGCGCGTTTTGACCATCT	3′ phosphor	[17]

The presence of the fimbrial adhesion gene (*daaC)* for diffusely adherent *E. coli* (DAEC)*,* the anti-aggregation protein transporter gene *(aat)* for enteroaggregative *E. coli* (EAggEC) heat-stable (*ST*) and heat-labile (*LT*) enterotoxin genes for enterotoxigenic *E. coli* (ETEC), the invasive plasmid antigen (*ipa*) gene for enteroinvasive *E. coli* (EIEC), the bundle-forming pili (*bfp*) gene for typical enteropathogenic *E. coli* (EPEC) and the intimin coding gene (*eae*) for EPEC were determined using end-point PCR on an Applied Biosystems™ 2720 Thermal Cycler platform using the QIAGEN PCR-multiplex kit (QIAGEN GmbH, Hilden, Germany) followed by agarose gel detection as previously described [19], using primers as shown in Table 2 (Inqaba Biotec Laboratory, South Africa).

**Table 2:** Primers for amplification of diarrheic *E. coli* virulence genes by conventional PCR

	Target genes	Primers	Primer sequence	Product size	Reference
A	*Eae*	*eae*-F	TCAATGCAGTTCCGTTATCAGTT	482 bp	[20, 21]
		*eae*-R	GTAAAGTCCGTTACCCCAACCTG		
	*Bfp*	*bfp*-F	GGAAGTCAAATTCATGGGGGTAT	298 bp	[21]
		*bfp*-R	GGAATCAGACGCAGACTGGTAGT		
	*stx*_*1*_	*stx*_*1*_-F	CAGTTAATGTGGTGGCGAAGG	348 bp	[22]
		*stx*_*1*_-R	CACCAGACAATGTAACCGCTG		
	*stx*_*2*_	*stx*_*2*_-F	ATCCTATTCCCGGGAGTTTACG	584 bp	[22]
		*stx*_*2*_-R	GCGTCATCGTATACACAGGAGC		
B	*Est*	*ST*-F	ATTTTTCTTTCTGTATTGTCTT	190 bp	[19]
		*ST*-R	CACCCGGTACAAGCAGGATT		
	*Elt*	*LT*-F	GGCGACAGATTATACCGTGC	440 bp	[19]
		*LT*-R	CGGTCTCTATATTCCCTGTT		
C	*Ipa*	*ipaH*-F	CTCGGCACGTTTTAATAGTCTGG	933 bp	[21]
		*ipaH*-R	GTGGAGAGCTGAAGTTTCTCTGC		
	*Aat*	*pCVD432*-F	CTGGCGAAAGACTGTATCAT	630 bp	[19]
		*pCVD432*-R	CAATGTATAGAAATCCGCTGTT		
	*daaC*	*daaC*-F	CAGGTCATCCGGTCAGTCGG	212 bp	[19]
		*daaC*-R	CAATGCCACGTACAACCGGC		

The positive control strains that were used for the different diarrheic *E. coli* pathotypes are shown below (Table 3) ([23]):

**Table 3:** Positive control strains used to test for diarrheic *E. coli*

Reference control strain	Virulence genes
*E. coli* ATCC 43887	*eae, bfp, wbdl*
*E. coli* C4193–1	*stx1, stx2, rfbE, hlyA, uidA*
*E. coli* H10407	*est, elt*
*E. coli* ATCC 43893	*ipa*
*E. coli* ATCC 3591–87	*aat*
*E. coli* D2190	*daaC*

Similarly, sample TSB enrichment broths from all sources were subjected to nucleic acid extraction using the MagNApure bacterial/fungal DNA extraction kit on a MagNApure LC instrument (Roche Diagnostics, Industriestrasse, Switzerland). PCR testing was done as described above [24].

*Shigella* were serotyped using the Wellcolex* colour *Shigella* Rapid latex agglutination test (Oxoid, Basingstoke, UK), while *Salmonella* and *E. coli* (O-typing) [25] were serotyped at the National Institute for Communicable Disease (NICD), Sandringham, Johannesburg using standard serological methods.

### Enumeration of faecal coliforms in water

Six ten-fold serial dilutions of 10 ml of canalized water in 90 ml of sterile water were filtered through 0.45 μM nitrocellulose membranes (Pall Corporation, Port Washington, USA) under vacuum suction and incubated on modified mTEC agar (Difco, Detroit, USA) at 35 °C for two hours and later at 44 °C for 22 h as previously described, followed by manual counting of colonies [16].

### Data analysis

Data on clinical and epidemiological characteristics were analyzed using Epi Info 7™ (CDC, Atlanta, USA). Comparison of proportions was done using a two-tailed Chi-square test or Fischer’s exact test, with *p* <  0.05 considered significant. MIC data were analyzed using the WHONET version 5.6 software (WHO, Geneva, Switzerland).

## Results

Stool specimens were collected from 66 children with diarrhoea (mean age ~ 15 months, range 2–36 months), 85 meat samples and 64 community canal water samples. Thirty-nine percent of children had received rotavirus immunisation. This study targeted patients three years and younger that presented with diarrhoea to the local centre during the study period. Among the children that presented with diarrhoea*,* 5% (3/66), 20% (13/66), and 17% (11/33) reported bloody stool, vomiting, and fever respectively (Table 4).

**Table 4:** Clinical and epidemiological characteristics of enrolled participants

Characteristics		Occurrence or duration
Sex	Male	30 (45, 95% CI = 34–57)
	Female	36 (55, 95% CI = 43–66)
Age range (months)		2–36
Mean duration of diarrhoea (±SD) days		2 (1.5)
Blood in stool (%)		3 (4.5, 95% CI = 1.6–13)
Vomiting (%)		13 (19.7, 95% CI = 12–31)
Fever (> 38 °C) (%)		11 (16.7, 95% CI = 10–27)
Weakness and dehydration (%)		30 (45.5, 95% CI = 34–57)
Cough (%)		6 (9.1, 95% CI = 4–18)
Belly pain (%)		3 (4.5, 95% CI = 1.6–13)
Cases on antibiotic therapy (%)		1(1.5, 95% CI = 0.3–8)

Overall, an enteric bacterial pathogen was isolated from 55% (36/66) of the cases; in 17% (6/36) of these, more than one bacterial pathogen was isolated (*Shigella flexneri* and STEC-one patient, *Shigella flexneri* and *Campylobacter jejuni-*one patient, *Aeromonas sobria* and DAEC-one patient, *Shigella flexneri* and DAEC-one patient, DAEC and EAEC-one patient, DAEC and EAggEC-one patient). There was a high prevalence of *S. flexneri* (Table 5) and DAEC (Table 6) in stool. Blood in stool was reported in cases where STEC (1/1), *A. sobria* (1/2), and DEC (1/14) were isolated. Notably, none (0/11) of the *S. flexneri* cases presented with bloody stool.

**Table 5:** Prevalence of food and waterborne pathogens in human and non-human sources in Nyanga

Isolates	Pathogens isolated per sample type n (%)		
	Stool (n = 66)	Meat (n = 85)	Water (n = 64)
*S. flexneri*	11 (17)	–	3 (5)
*P. shigelloides*	7 (9)	1 (1)	11 (17)
*S. enterica*	4 (6)	4 (5)	–
*C. jejuni*	3 (5)	6 (7)	–
*A. sobria*	2 (3)	3 (3)	4 (6)
*V. vulnificus*	–	–	6 (9)

**Table 6:** Diarrheic *E. coli* isolated on CHROMagar™STEC versus corresponding PCR target genes detected in the respective sample type enrichment

PCR targets	Stool (66)		Meat (85)		Water (64)	
	Isolates	Genes	Isolates	Genes	Isolates	Genes
EaggEC / *aat*	2 (3)	9 (14)	–	–	1 (2)	–
DAEC/*daaC*	8 (12)	23 (35)	2 (2)	6 (7)	2 (3)	24 (38)
STEC/*stx*_*1*_	1 (2)	4 (6)		17 (20)	1 (2)	19 (30)
STEC/*stx*_*2*_	–	2 (3)	–	4 (5)	–	–
EIEC/*ipa*	1 (2)	4 (6)	–	–	–	5 (8)
ETEC/*LT*	–	5 (8)		1 (1)	–	21 (33)
O157/*rfbE*	–	1 (2)		5 (6)	–	9 (14)
O111/*wbdl*	–	1 (2)		–	–	5 (8)
EPEC/*eaeA*	–	–	1 (1)	6 (7)	–	–

The various raw meat samples (chicken, beef, pork, and mutton) that were tested carried various bacterial pathogens which included diarrheic *E. coli*, *C. jejuni*, *S. enterica* serotype Idikan, *A. sobria* and *P. shigelloides*. None of the targeted bacterial pathogens were isolated from any of the roasted meat samples. No tellurite resistant STEC, EAggEC, ETEC, DAEC or EIEC were isolated from any of the meat samples in this setting (Table 6).

**Fig. 2:**
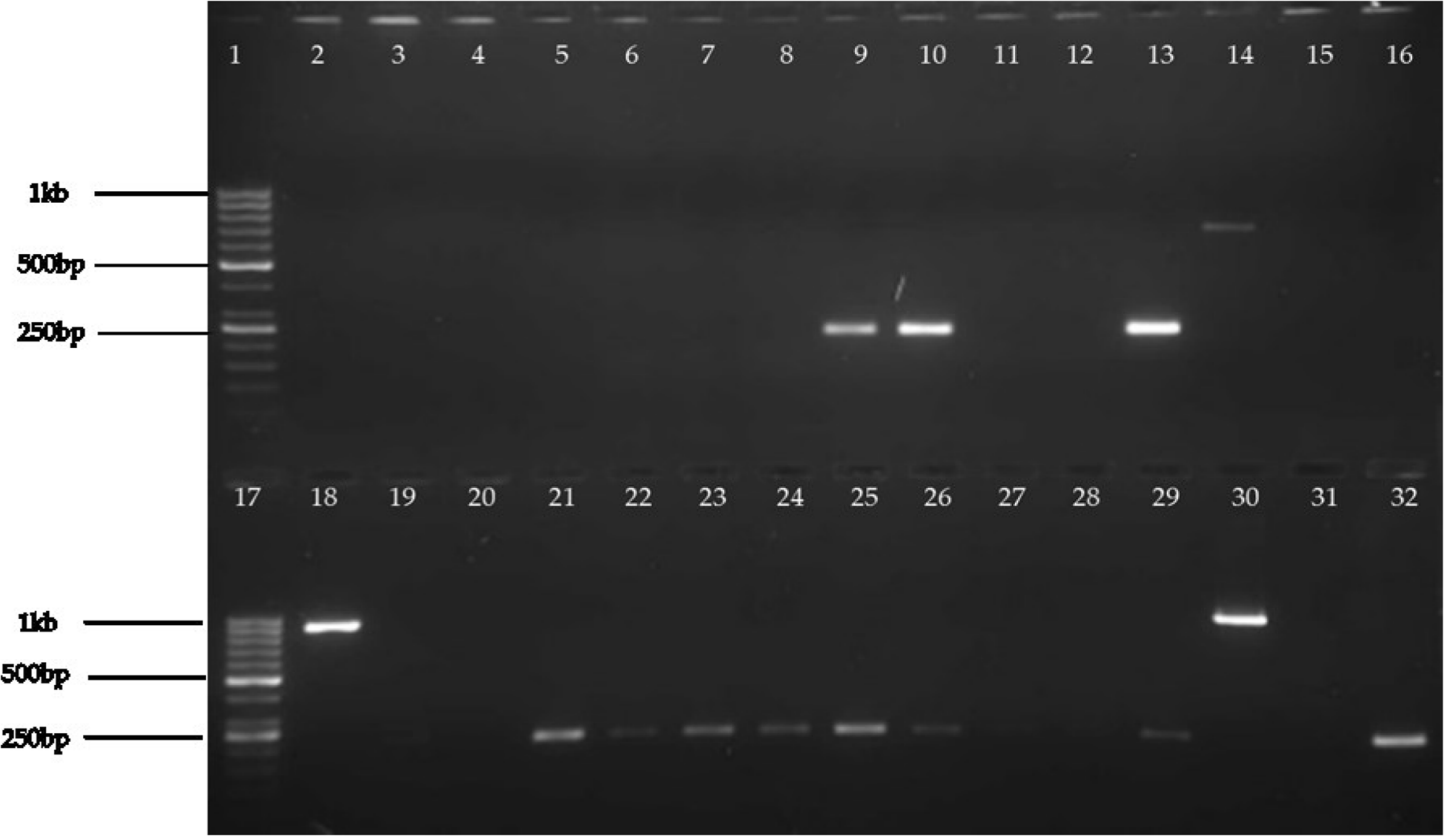
Electrophoresis gel image for PCR product following multiplex PCR ampification for *aat*, *ipa*, and *daaC*. Lanes 1 and 17 contained the 1 kb ladder (GeneRuler™ DNA molecular weight ladder -ThermoFisher Scientific Inc., MA, USA). Lanes 2–32 contained samples except for lanes 14 (positive control for *aat*),18 (positive control for *ipa*), 19 (negative control), 32 (positive control for *daaC*). Samples in lanes 9, 10, 13, 21, 22, 23, 24, 25, 26, and 29 were positive for *daaC*. The sample in lane 30 was positive for *ipa*. The rest of the samples were negative for *aat*, *ipa*, and *daaC*. The gel image was captured using the E-Gel GelCapture Software (version 1.7)

For all the pathotypes, more of the virulence marker genes were detected (Fig. 2) in TSB enrichment of stool as compared to the actual number of diarrheic *E. coli* pathotypes isolated on CHROMagar™STEC. This could be explained by the fact that not all diarrheic *E. coli* are tellurite resistant and so would not form colonies on CHROMagar™STEC.

Nevertheless, there was a high prevalence of *stx*_*1*_ (20%, 17/85) in TSB enrichment broths of these samples. For raw beef, *S. enterica*, *P. shigelloides*, *A. sobria*, and EPEC were isolated. For raw pork, only DAEC was isolated while *S. enterica* and *C. jejuni* were isolated from raw chicken (Table 7).

**Table 7:** Distribution of bacterial pathogens in the different meat types from the informal meat trade in Nyanga

Type (n)	Number of samples with virulence gene/ bacterial pathogen (%)				
	*Salmonella*	*Campylobacter*	*Plesiomonas*	*Aeromonas*	DEC
Raw beef (17)	1 (6)	–	1 (6)	2 (12)	2 (12)
Raw mutton (9)		1 (11)	–	1 (11)	–
Raw pork (22)		–	–	–	1 (5)
Raw chicken (11)	3 (27)	5 (45)	–	–	–
Roast beef (26)		–	–	–	–
Roast Pork (8)		–	–	–	–
Roast Chicken (3)		–	–	–	–
Total	4	6	1	3	3

DEC = Diarrheic *E. coli*,” – “means that the pathogen was not isolated

**Fig. 3:**
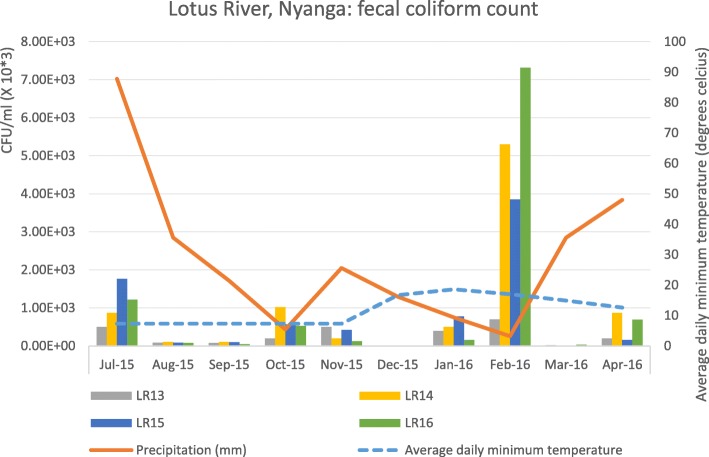
Mean faecal coliform counts (CFU/ml) for four water collection points along the Lotus River (LR13, LR14, LR 15, and LR 16), monthly average rainfall (mm), and average daily minimum temperatures (°C) between July 2015 and April 2016. The Figure was developed using the Microsoft Office Excel 365 ProPlus, version 1902

Surface water from the community canal (at sampling points LR13, LR14, LR15, and LR16) yielded high numbers of faecal coliforms (mean CFU/ml =2.74E+ 05–1.11E+ 06) with the highest levels seen in February (p = < 0.001), which was also the driest (zero precipitation) and hottest month; precipitation in March 2016 coincided with the lowest measured surface water contamination level (Fig. 3). *P. shigelloides* (11/64, 17%) was the most abundant enteric bacterial pathogen in water; others included *S. flexneri*, *V. vulnificus*, *A. sobria*, EAggEC, STEC, and DAEC. The samples, however, had a high prevalence of *daaC* (38%, 24/64) and *stx*_*1*_ (30%, 19/64).

For all sample types, diarrheic *E. coli* virulence genes were detected more frequently with direct testing from TSB, compared to isolation from CHROMagar™STEC. Of note, all isolates on CHROMagar™STEC yielded corresponding PCR targets on direct TSB PCR.

### Antimicrobial susceptibility patterns of diarrheic *E. coli* and other food and waterborne pathogens

All the pathogens from the different sources were resistant to trimethoprim**-**sulfamethoxazole. Overall, amongst the 20 diarrheic *E. coli* isolates (used as sentinels for antimicrobial resistance surveillance), there was resistance to the following antibiotics: ampicillin, amoxicillin-clavulanate, cefoxitin, cefuroxime, cefotaxime, cefepime, ciprofloxacin, trimethoprim-sulfamethoxazole, and nitrofurantoin (Table 8).

**Table 8:** Sources, pathotypes, serotypes, and resistance profiles of diarrheic *E. coli* in Nyanga

Isolate Number	Source	Pathotype	Serotype	Resistance profile
777	Mutton	DAEC	Non-Typeable	AMP AMC CXM CXA
LR16	Water	DAEC	O101	AMP
NY29	Child	STEC	O106	AMP
789	Beef	EPEC	Non-Typeable	–
NY3.2	Child	DAEC	Non-Typeable	–
NY1.2	Child	DAEC	O153	AMP AMC CXM CXA FOX CTX
NY50	Child	EPEC	O49	–
767	Water	DAEC	Non-Typeable	AMP AMC TZP
NY1.1	Child	DAEC	O153	AMP AMC CXM CXA FOX CTX
NY13.1	Child	EaggEC	Non-Typeable	–
NY43	Child	EaggEC	O143	–
NY58	Child	DAEC	Non-Typeable	–
710	Water	STEC	Non-Typeable	–
NY4	Child	EIEC	Non-Typeable	–
E101.1	Water	EaggEC	Non-Typeable	–
NY60	Child	DAEC	Non-Typeable	AMP
NY28	Child	DAEC	Non-Typeable	AMP
E33.1	Child	DAEC	Non-Typeable	–
E34.1	Child	DAEC	Non-Typeable	–
PK-STEC	Pork	DAEC	Non-Typeable	–

Expert interpretation rules embedded in the WHONET software were used to classify as resistant or susceptible. AMP = ampicillin, AMC = amoxicillin-clavulanate, CTX = cefotaxime, TZP = tazobactam-piperacillin, CXM = cefuroxime, CXA = cefuroxime axetil, FOX = cefoxitin

Only three isolates were multi-drug resistant and were isolated from meat (one isolate) and stool (two isolates). All *Plesiomonas* (from water and stool) and *Aeromonas* (from meat and stool) were resistant to ampicillin while all *Salmonella enterica* Idikan and *Salmonella enterica* non – typeable were resistant to ciprofloxacin (Table 9).

**Table 9:** Percentage non-susceptible per specimen type for *Salmonella*, *Shigella*, *Plesiomonas*, *Vibrio*, and *Aeromonas*

Antibiotic	*Salmonella* n (%R)		*Shigella* n (%R)		*Plesiomonas* n (%R)		*Aeromonas* n (%R)			*Vibrio* n (%R)
	Meat (n = 4)	Stool (*n* = 4)	Water (n = 4)	Stool (n = 11)	Water (*n* = 11)	Stool (n = 6)	Meat (*n* = 3)	Stool (*n* = 2)	Water (n = 4)	Water (n = 6)
AMP	–	–	3 (75)	7 (64)	11 (100)	6 (100)	3 (100)	2 (100)	–	–
AMC	–	–	3 (75)	7 (64)	–	–	–	–	–	–
CXM	4 (100)	1 (25)	–	6 (55)	–	–	–	–	–	–
CXA	1 (25)	–	1 (25)	6 (55)	–	–	–	–	–	–
FOX	4 (100)	1 (25)	–	8 (73)	2 (18)	–	–	–	–	6 (100)
CAZ	–	–	–	–	–	1 (17)	–	–	–	–
ETP	–	–	–	–	–	–	3 (100)	2 (100)	–	6 (100)
AMK	4 (100)	–	–	7 (64)	–	–	–	–	–	1 (17)
GEN	1 (25)	1 (25)	–	7 (64)	2 (18)	–	–	–	–	–
CIP	4 (100)	4 (100)	–	–	–	–	–	–	–	–
TGC	–	–	–	–	–	–	–	2 (100)	–	–
SXT	4 (100)	4 (100)	4 (100)	11 (100)	11 (100)	6 (100)	3 (100)	2 (100)	4 (100)	6 (100)

*NT = Not Tested for. *Campylobacter* from water, stool, and the meat was all susceptible to ciprofloxacin and thus not included in this table. Expert interpretation rules embedded in the WHONET software were used to classify as resistant or susceptible. The CLSI clinical guidelines and breakpoints were used to analyse the data sets. AMP = ampicillin, AMC = amoxicillin-clavulanate, CXM = cefuroxime, CXA = cefuroxime axetil, FOX = cefoxitin, AMK = amikacin, GEN = gentamicin, CIP = ciprofloxacin, SXT = trimethoprim-sulphamethoxazole, NIT = nitrofurantoin

## Discussion

This study revealed a high prevalence of food and waterborne, as well as sanitation associated bacterial pathogens in meat, surface water and diarrheal stool from a low-income informal settlement setting in Cape Town, South Africa. Assessment of non-human sources for diarrheic bacterial pathogens is important because environmental modifications of potential reservoirs would aid the prevention of infections with drug-resistant pathogens.

Among the children that presented with diarrhoea*,* 5% (3/66), 20% (13/66), and 17% (11/33) reported bloody stool, vomiting, and fever respectively. Among the bacterial pathogens detected, *S. flexneri*, *C. jejuni*, STEC, and EIEC have been commonly reported to cause invasive disease. The associations between the presence of these bacterial pathogens and the different clinical presentations could not be determined due to small sample size. Notably, none (0/11) of the *S. flexneri* cases presented with bloody stool - a finding consistent with previous studies that reported the poor sensitivity of this clinical sign for the recovery of Shigellae [9, 10]. *S. flexneri* was the second most prevalent pathogen (the highest was DAEC) in this setting; it was the most prevalent among systemic shigellosis cases diagnosed between 2003 and 2009 in South Africa [26]. *Shigella* and other dysentery-causing infections may be underappreciated in primary health care in South Africa. Appropriate laboratory testing and surveillance to support the differential diagnosis and targeted treatment of enteric infections in vulnerable communities are urgently needed to aid patient care and reduce secondary transmission in the community. At least seven outbreaks of shigellosis or bacillary dysentery have been reported from South Africa since 1994. These have been associated with poor water and sanitation infrastructure. Our study community experienced an outbreak of bacillary dysentery in 1999 that affected 82 people and resulted in 3 deaths in children under two years of age [27]. Without access to laboratory testing, many cases will go undetected hence resulting in poor patient outcomes and increased health expenditure.

Similar studies in South Africa, albeit conducted in non – informal settlement settings, have reported a prevalence of 10% for *Campylobacter* (compared to 5% in this study) in the stool in the Venda region, Limpopo, South Africa [28]. Among the diarrheic *E. coli* that were detected in stool in this setting, DAEC were the most common (12%, 8/66 for isolates and 23%, 35/66 for marker genes) and EIEC were the least common (isolates 2%, 1/66 and marker genes 6%, 4/66). An earlier report in Egypt, 2013, established presence of DAEC associated with diarrhoea in human and non-human sources [29]. Our study highlights DAEC as a diarrhoea associated foodborne pathogen in this informal settlement setting. Even though EIEC is thought to be endemic in Africa settings causing traveller’s diarrhoea, our findings have shown that it was not prevalent in this local population. For all the diarrheic *E. coli* that were detected, more of the virulence marker genes were detected by these methods, compared to the isolates on CHROMagar™STEC from the same sample. Infection with multiple organisms was common (17% - 6/36 of the stool samples that carried a bacterial pathogen). All co-infected patients were infected with at least one of *S. flexneri* or DAEC. We did not test for infection or co-infection with viruses or intestinal parasites. Consistent with the reported inadequate and low second rotavirus vaccine coverage in the Cape Town metro [6] only 39% (26/66) of our cohort reported receiving rotavirus vaccination.

Most of the cases (79% - 52/66) used the communal standpipe as the primary source of water. According to the City of Cape Town Water and Sanitation Department, the communal standpipe nozzles in the informal settlements often were contaminated [30]. Lack of access to clean water is linked to high incidences of diarrheal disease [31].. Faecal contamination was demonstrated by the persistent significantly high levels of faecal coliforms in the local water canal that is accessible to domestic animals and humans. The increased level of contamination seen in the canal during the hottest and driest month of February (zero precipitation) is informative to aid waterborne disease risk management and public information, particularly during a period when human and animal exposure is more likely. The recovery of viable *S. flexneri* from open surface water in the community is alarming as Shigellae are particularly evanescent outside the host, suggesting a substantial presence. *P. shigelloides* and *V. vulnificus* were abundant in canal water raising concern for those accessing the canal for recreation, washing, food extraction or drinking; and to those executing monitoring activities. The prevalence of *stx*_*1*_ genes in water (30% of samples) was higher than the 15% previously detected in the Berg River system [32]. The presence of *rfbE* and *wbdl* in the water highlights it as a potential reservoir of STEC O157: H7 and STEC O111. This is of clinical and epidemiological significance because these are highly pathogenic STEC serotypes [33].

Raw meat samples collected from informal street-side vendors showed *C. jejuni* and *S. enterica* Idikan were most common pathogens identified. Of concern, *A. sobria* and *P. shigelloides*, typically found in aquatic habitats, were recovered from meats, raising concern over vendor access to safe water and sanitation infrastructure. *S. enterica* Idikan was isolated from 5% (4/85) of the meat samples. It has previously been isolated from food-producing animals; animal feeds, meat from abattoirs, water, and associated environments in South Africa at a prevalence of 6% (between 2012 and 2014) [34]. *C. jejuni* in South Africa is commonly isolated from poultry and is increasingly becoming resistant to antibiotics [35–37]. The microbiological standards for foodstuffs sold in South Africa regard the recovery of *Salmonella*, *Shigella* or *E. coli* of any quantity as unacceptable [38].

Considering diarrheic *E. coli* as sentinels for surveillance of foodborne antimicrobial resistance in this setting, these pathogens showed resistance to antibiotics usually used as first-line options for treatment of infections caused by Gram negative bacilli, such as ampicillin, amoxicillin-clavulanate, and extended-spectrum cephalosporins.

### Limitations of this study

Pathogen-specific enrichment methods would have been more sensitive for isolate recovery. Viral and parasitic causes of diarrhoea were not targeted in this study.

## Conclusions

We showed that antimicrobial resistance to a number of antimicrobials is present in diarrheal pathogens from human, food, and environmental sources in an informal settlement in Cape Town.*S. flexneri* spreads from person-to-person as a result of poor sanitation and inadequate quantities of water for personal hygiene, highlighting the need to urgently address inadequate water and waste provision in communities such as this one.

Globally, informal settlements present unique public health challenges due to dense human populations, poor residential infrastructure, and poor hygiene practices. Informal abattoirs do not follow recommended slaughter practice and often contaminate the environment. In a setting where diarrhoea is often empirically managed, knowledge of the sources, prevalence and antibiotic susceptibility patterns of bacteria causing enteric infections is critical.

## Data Availability

All data generated or analysed during this study are included in this published article. The datasets used and/or analyzed during the current study are available from the corresponding author on reasonable request.

## Abbreviations

*aat*: Anti: aggregation protein transporter gene
AMK: Amikacin
ATCC: American Type Culture Collection
AZT: Aztreonam
*bfp*: Bundle forming pilus
CAZ: Ceftazidime
CDC: Centre for Disease Control and Prevention
CIN: Gentamicin
CIP: Ciprofloxacin
CLSI: Clinical Laboratory Standards Institute
COL: Colistin
CTX: Cefotaxime
*daaC*: Fimbrial adhesion gene
DAEC: Diffusely adherent *Escherichia coli*
DOR: Doripenem
DOX: Doxycyclin
*eae*: intimin gene
EAggEC: Enteroaggregative *Escherichia coli*
EIEC: Enteroinvasive *Escherichia coli*
EPEC: Enteropathogenic *Escherichia coli*
ETEC: Enterotoxigenic *Escherichia coli*
ETP: Ertapenem
F: Female
FEP: Cefepime
HUS: Haemolytic Uremic Syndrome
IMP: Imipenem
*ipa*: invasive plasmid antigen
LEV: Levofloxacin
LT: Heat labile enterotoxin gene
M: Male
MEM: Meropenem
MIC: Minimum inhibitory concentration
MIN: Minocycline
NHLS: National Health Laboratory Services
PCR: Polymerase chain reaction
POL: Polymixin
ST: Heat stable enterotoxin gene
STEC: Shiga toxin producing *Escherichia coli*
Stx: Shiga toxin gene
SXT: Trimethoprim sulfamethoxazole
Ter: Tellurium resistance gene
TGC: Tigecycline
TIM: Ticarcillin clavulanic acid
TOB: Tobramycin
TSB: Tryptic soy broth
TZP: Piperacillin-Tazobactam
